# Supplementary material for: Sample Size Calculations in Simple Linear Regression: A New Approach
Source: Entropy (Basel). 2023 Apr 3;25(4):611. doi: 10.3390/e25040611 (PMC10137477; doi:10.3390/e25040611)
Supplement: Supplementary file 1 [file entropy-25-00611-s001.zip › entropy-2262474-supplementary-File S2.pdf]

Supplementary Material S1

Null Distribution of T^2

Simulations and R Code

The distribution of T^2 is determined by sampling $W_1, W_2, W_3, and\ W_4$ extensively. Any sampling would incur sampling errors. To have a leverage on the errors, the entire process of getting the critical values is carried out 1000 times. This will provide the mean and the standard deviation of the critical values. A panoramic view of sampling and attendant calculations:

Step 1: Fix n and α.

Step 2: Simulate $W_1 \sim \chi_1^2$.

Step 3: Simulate $W_2 \sim \chi_{n-1}^2$.

Step 4: Simulate $W_3 \sim \chi_{n-2}^2$.

Step 5: Simulate $W_4 \sim \chi_{n-1}^2$.

Step 6: Form the ratio $\frac{(n-2)}{(n-1)} * \frac{W_1 W_4}{W_2 W_3}$

Step 7: Repeat steps 2, 3, 4, 5 and 6 10,000 times.

Step 8: Calculate the $(1-\alpha) * 100^{\text{th}}$ percentile $C_{n,\alpha}^2$ of the ratios.

Step 9: Calculate the positive square root (critical value) $C_{n,\alpha}$ of $C_{n,\alpha}^2$.

Step 10: Repeat Step 9 1000 times.

Step 11: Calculate the mean and standard deviation of $C_{n,\alpha}$.

Step 12: Record the results average $(C_{n,\alpha})$ and SD in Table 1.

The R code is given below.

Calculate Critical Values, Average of Critical Values, and Standard Deviations

Set the level of significance.

```
Alpha <- 0.01
```

Simulate the distribution of T^2 for each of n = 20 (1) 100 by generating 10,000 values from the distribution. The (1 – Alpha)-th percentile of the data is the squared critical value $C_{n,\alpha}^2$ Repeat the process 1,000 times. We use the loop function in R.

Create a matrix of zeros with 81 rows and 1000 columns. We will populate the matrix with $C_{n,\alpha}$ s column by column 1,000 times. The rows represent n.

- Sample=matrix(0,81,1000)

Start a loop to populate Sample.

- for (i in 1:81)
- {
-   for (j in 1:1000)
- {

  # $C_{n,\alpha}$ s are computed.

- Sample [i,j]<-sqrt(quantile(((i+19-2)/(i+19-1))*rchisq(10000,1)*rchisq(10000,i+19-1)/(rchisq(10000,i+19-1)*rchisq(10000,i+19-2)),0.99))
- }
- }

We calculate the mean of $C_{n,\alpha}$ s for each n over 1,000 repetitions.

- Mean1=rowSums(Sample)/1000

We calculate the standard deviation of 1,000 $C_{n,\alpha}$ s for each n. We use another loop.

- SD1=rep(0,81)
- for (i in 1:81)
- {
- SD1[i]<-sd(Sample[i,])
- }

The means and standard deviations are rounded to three and four decimal places, respectively.

- Mean0.01=round(Mean1,3)
- SD0.01=round(SD1,4)

These two columns are uploaded into Table 1 under the level = 0.01.

For the results on the levels 0.05 and 0.10, make changes appropriately in the first loop in the quantile function.

Supplementary Material S2

Power and Sample Size Calculations

Simulations and R Code

For power calculations, we have resorted to simulations. We generate data from the regression model. Our strategy is as follows.

a. Spell out $\delta = \beta_1 * \sigma_X/\sigma$.
b. Take $\sigma_X = 1$, and $\sigma = 1$. We can set these values at the numbers specified, because the alternative distribution depends only on the effect size.
c. For given n, draw a random sample $X_1, X_2, \ldots, X_n$ of size n from N (0, 1).
d. For each $1 \leq i \leq n$, draw a random sample of size 1 from $N(\beta_1 * X_i, 1)$.
e. We thus have the data: $(X_1, Y_1), (X_2, Y_2), \ldots, (X_n, Y_n)$ from the model X ~ N (0, 1) and Y | X ~ $N(\beta_1 * X, 1)$. We are taking $\mu_X = 0$ and $\beta_0 = 0$. They do not play any role at all in the distributions identified.
f. Estimate β_1, σ_X, and σ.
g. Calculate $T = \hat{\beta}_1 * \hat{\sigma}_X/\hat{\sigma}$.
h. Check $|T| > C_{n,\alpha}$ with $C_{n,\alpha}$ coming from Table 1.
i. Set up a counter = 1 if $|T| > C_{n,\alpha}$, = 0, otherwise.
j. Repeat Steps a to i one-thousand times.

k. Calculate Power = $(\# \, Counter = 1)/1000$.

l. If Power matches the targeted power 1 – β, stop. Otherwise, keep experimenting with n until the targeted power is attained.

m. Once the sample size n is found out, we want to make sure that this is the right number. For the determined n, repeat Step a to l one thousand times. The average power and standard deviation are also reported.

n. Tables 2, 3, and 4 embody our effort under this strategy.

o. The R code is given below.

Sample size calculations.

The levels of Alpha (0.01, 0.05, 0.10), Power 1 – Beta (80%, 90%, 95%, 99%), and Effect Size (ES) (0.1 (0.1) 0.6) are set. For each combination of Level, Power, and ES, we start with an arbitrary sample size n, simulate the distribution of T^2 under the prescribed environment, and check whether the power is attained. If not, we tweak the sample size judiciously until the power is attained. This is labor intensive. A sophisticate in R can simplify our process considerably.

Create an R folder Sample1 of 81 rows (sample sizes n = 20 (1) 100) and three columns (Alpha = 0.01, 0.05, 0.10) storing the critical values.

Simulate the simple linear regression model.

Set $\beta_0 = 0$, $\mu_X = 0$, ES = 0.1. The standard deviations are taken to be each equal to unity. The slope parameter $\beta_1 = 1$. Generate n observations from the model. Estimate the slope parameter and variances. Calculate the value of the test statistic T. Check whether the test statistic exceeds the critical value in absolute value. Repeat the whole process 1,000 times. Calculate the power empirically. In the following, we set Alpha = 0.05.

Specifications for X and Y: X ~ N(0, 1) and Y | X ~ N(0.1*X, 1)

<u>Code A</u>

Create a function of the sample size n.

➢ Sample2<-function(n){

Simulate the distribution of X n times and repeat it 1,000 times.

➢ Tra1<-matrix(rnorm(n*1000,0,1),nrow=n,ncol=1000)

➢ Tra2<-matrix(0,nrow=n,ncol=1000)

Simulate the conditional distribution of Y.

➢ for(i in 1:n){ for(j in 1:1000){

➢ Tra2[i,j]<-rnorm(1,0.1* Tra1[i,j],1)

➢ }}

Fit the simple linear regression model for each n-data. Store the estimates of the slope parameter.

➢ Tra3<-rep(0,1000)

➢ for (j in 1:1000){

➢ Tra3[j]<-(lm(Tra2[,j]~ Tra1[,j]))$coefficients[2]}

Estimate the standard deviation of X for each n-data and store the results.

➢ Tra4<-rep(0,1000)

➢ for (j in 1:1000){

➢ Tra4[j]<-sd(Tra1[,j])}

Estimate the conditional variance for each n-data and store the results.

➢ Tra5<-rep(0,1000)

➢ for (j in 1:1000){

➢ Tra5[j]<-sqrt((sum((lm(Tra2[,j]~ Tra1[,j]))$residuals^2))/(n-2))}

Calculate the value of the test statistic T for each n-data and store the results.

➢ Tra6<-rep(0,1000)

➢ Tra6<-((Tra3* Tra4)/ Tra5)

Open a counter to check whether the test statistic exceeds the critical value.

➢ Tra7<-rep(0,1000)

- for(j in 1:1000){
- Tra7[j]<-ifelse(abs(Tra6[j])>Sample1[n-19, 2],1,0)}
- power<-sum(Tra7)/1000
- return(power)}

This function of n will give the empirical power for any n for Alpha = 0.05 for the simple linear regression model set at the outset. For other levels, make changes in Sample1in the third line from the bottom of Code A.

Note:

All these computational steps will calculate the power for a given n and $\alpha = 0.05$. The power may not be equal to the targeted power 1-β. Experiment with n until the targeted power is reached. This is the most labor-intensive maneuver requiring hundreds of hours of work. After this monumental work, we will have tables of sample sizes for every choice of α =0.01, 0.05, 0.1, 1-β = 80%, 90%, 95%, 99%, and Effect Sizes= 0.1, 0.2, 0.3, 0.4, 0.5, 0.6.

A. Verifying whether the sample size determined in B and reported in Tables 2, 3, and 4 is the right one.

Set Alpha, Power, ES, and the sample size n reported in Tables 2, 3, or 4 as the case may be. Calculate the power following the code in B. Repeat it 1,000 times.

- La<-rep(0,1000)
- for (i in 1:1000)
- {
- La[i]<-Sample2(790)
- }

mean(La)

sd(La)

These numbers are reported in Tables 2, 3, and 4 for each of the choices of α, $1-\beta$, and ES. Make sure that the sample size n is correctly reported in Sample2(n). This is also labor-intensive.
